# Ventilation strategy has a major influence on remote ischaemic preconditioning in mice

**DOI:** 10.1111/jcmm.13164

**Published:** 2017-04-04

**Authors:** Sean M. Davidson, Zhenhe He, Alex Dyson, Daniel I. Bromage, Derek M. Yellon

**Affiliations:** ^1^ The Hatter Cardiovascular Institute University College London London UK; ^2^ Magnus Sciences University College London London UK

**Keywords:** ischaemia, reperfusion, ventilation, oxygen, cardioprotection, preconditioning, remote preconditioning, bradykinin, blood flow, reactive hyperaemia

## Abstract

Whether oxygen should be administered acutely during ST‐segment elevation myocardial infarction is debated. Despite this controversy, the possible influence of supplementary oxygen on animal models of ischaemia–reperfusion injury or cardioprotection is rarely considered. We used an *in vivo* mouse model of ischaemia and reperfusion to investigate the effect of ventilation with room air *versus* 100% oxygen. The coronary artery of anaesthetized mice was occluded for 40 min. followed by 2‐hrs reperfusion. Infarct size was measured by tetrazolium staining and expressed as a percentage of area at risk, determined using Evan's blue. Unexpectedly, infarct size in mice ventilated with 100% oxygen was significantly smaller than in those ventilated with room air (33 ± 5% *versus* 46 ± 3%; *n* = 6; *P* < 0.01). We tested a standard protocol of 3 × 5 min. cycles of remote ischaemic preconditioning (RIPC) and found this was unable to protect mice ventilated with 100% oxygen. RIPC protocols using 2.5‐ or 10‐min. occlusion were similarly ineffective in mice ventilated with oxygen. Similar disparate results were obtained with direct cardiac ischaemic preconditioning. In contrast, pharmacological protection using bradykinin administered at reperfusion was effective even in mice ventilated with 100% oxygen, reducing infarct size from 33 ± 5% to 21 ± 3% (*n* = 4–6; *P* < 0.01). Laser speckle contrast imaging of blood flow and direct pO_2_ measurements were made in the hindlimb, but these measurements did not correlate with protection. In conclusion, ventilation protocol can have a major influence on infarct size and ischaemic preconditioning protocols in mice.

## Introduction

Oxygen is often administered to patients experiencing uncomplicated ST‐segment elevation myocardial infarction (STEMI). However, whether it is beneficial in this context remains controversial 1, 2. Remote ischaemic pre‐ and post‐conditioning (RIC) has been investigated as a method of protecting the myocardium against ischaemia and reperfusion injury that occurs during STEMI treated by primary percutaneous coronary intervention or during cardiac surgery 3, 4. Despite initial promise in the setting of coronary artery bypass graft (CABG) surgery, the outcomes of two recent large clinical trials were neutral 5, 6. Pilot studies of RIC in the setting of STEMI have shown more consistent benefit and large clinical trials are currently underway 7. However, it is important to recognize that although a number of factors have been proposed to influence the efficacy of RIC, few of these have been systematically examined in either the clinical or experimental setting. This fact is highlighted by a recent meta‐analysis that demonstrated a strong, consistent beneficial effect of RIC in reducing infarct size in *in vivo* animal experiments of ischaemia and reperfusion injury, but was unable to identify experimental variables that significantly contributed to the observed large experimental heterogeneity 8. In this meta‐analysis, the variable most strongly related to the degree of protection by RIPC (albeit not reaching significance), was the oxygenation status, that is to say whether animals were ventilated with room air or with high‐flow supplemental oxygen 8. This led us to hypothesize that RIPC in animal experiments might be affected by supplemental oxygen because limb pO_2_ takes longer to reach a sufficient hypoxic threshold for cardioprotection, a variable which has not been considered previously in such experiments.

To investigate the effect of ventilation strategy on RIPC in the experimental setting, and whether the duration of limb ischaemia is an important variable in the development of RIPC, we performed a set of infarction experiments in mice. In parallel, we measured limb pO_2_ during the RIPC procedure using a phosphorescent pO_2_ probe, as well as blood flow by laser speckle contrast imaging. These experiments revealed a number of unexpected observations which we believe are useful in understanding the role of ischaemic time and oxygenation in RIPC protocols in mice.

## Materials and methods

All animals received humane care in accordance with the United Kingdom Home Office Guide on the Operation of Animal (Scientific Procedures) Act of 1986. The investigation conforms to the guidelines from Directive 2010/63/EU of the European Parliament on the protection of animals used for scientific purposes or the NIH guidelines. All experiments approved by the appropriate ethics committee and have therefore been performed in accordance with the ethical standards laid down in the 1964 Declaration of Helsinki and its later amendments.

C57Bl/6 mice were anaesthetized by i.p. injection of 80 mg/kg pentobarbitone at a concentration of 20 mg/ml in 0.9% (w/v) saline and maintained at 36.5 ± 0.5°C on a heating mat. Surgery was started once pedal and tail reflexes were abolished and depth of anaesthesia was monitored throughout. Mice were intubated using a 19G cannula and ventilated using a MiniVent, type 845, Small Animal Ventilator (Harvard Apparatus, Kent, UK), supplemented with either room air or 100% oxygen, at a flow rate of 1.0 l/min. with 2 cmH_2_O PEEP, stroke volume 200 μl at 130 strokes/min. The left anterior descending (LAD) coronary artery then was occluded (verified by ST elevation, hypokinesia and pallor) for 40 min. followed by 2 hrs reperfusion, after which infarct size was measured by tetrazolium staining and expressed as a percentage of area at risk, determined using Evan's blue. A total of 82 mice were used for infarct experiments and were randomly assigned to treatment group. Seven mice died during reperfusion and were excluded from analyses (two control, one 5‐min. RIPC, two 10‐min. RIPC, one vehicle, one bradykinin).

**Figure 1:**
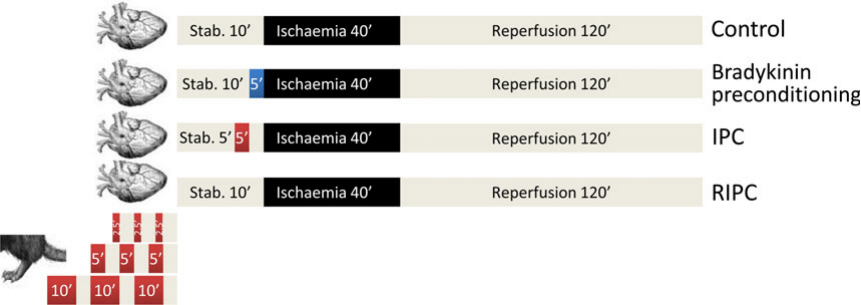
Experimental protocols. All mice were subject to 40‐min. coronary artery ligation followed by reperfusion for 120 min. *in vivo*. Preconditioning periods of ischaemia were applied either directly to the heart (‘classical’ IPC) or remotely, by inflation of a cuff on the limb (RIPC), and are indicated in red. 40 μg/kg bradykinin was administered i.v. 5 min. before ischaemia (blue).

RIPC was induced using a 6‐mm lumen custom vascular occluder (Kent Scientific, Torrington, CT, USA) around the right hindlimb inflated to 250 mmHg to induce three cycles of 2.5‐, 5‐ or 10‐min. ischaemia, followed by 5‐min. reperfusion after each cycle (Fig. 1). Classical, ‘direct’ ischaemic preconditioning (IPC) was induced by one cycle of 5‐min. LAD occlusion and 5‐min. reperfusion. Pharmacological preconditioning using bradykinin was administered by slow injection *via* the external jugular vein at 40 μg/kg in a vehicle of 0.1 mM acetic acid in saline, prior to index ischaemia. Vehicle control mice received 0.1 mM acetic acid in saline. Animals were killed by severing of the aorta.

Blood flow was visualized in the hindlimb during RIPC using a FLPI‐2 laser speckle contrast blood flow imager (Moor Instruments, Axminster, United Kingdom), which delivers images at high time and spatial resolution. Hair was first removed from both hindlimbs using Veet depilation. Full‐frame images were recorded at 5 Hz and then subsequently analysed by averaging the image intensity over an area of interest encompassing the entire exposed upper limb. Similar results were obtained if a region containing only the femoral artery or only smaller vessels was used.

Limb muscle pO_2_ was measured every 5 sec. using a bare‐fibre phosphorescent sensor connected to an Oxylite™ monitor (Oxford Optronix, Abingdon, United Kingdom). The probe was pre‐coated with a 10 U/ml solution of heparin in saline to prevent haematoma, then slowly inserted into the vastus intermedius muscle along the track of a puncture made using a 21G × 5/8” Microlance needle to a depth of 5 mm. tPO_2_ measurements were continuously recorded using PowerLab 4/25 coupled to Chart 7 (AD Instruments, Oxon, UK).

The results are shown as mean ± standard deviation of the mean. Statistical comparison of the groups was made by two‐way anova, with Bonferroni correction for multiple comparisons. A significance value of *P* < 0.05 was considered significant.

## Results

**Figure 2:**
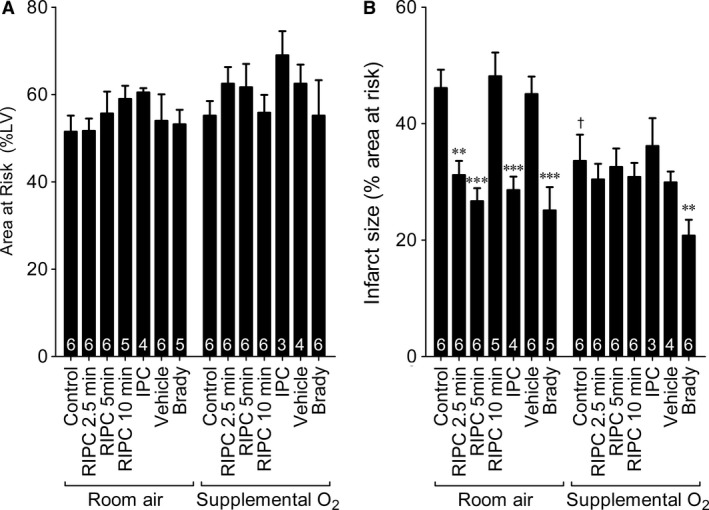
Infarct size after ischaemia and reperfusion compared in mice ventilated with room air or supplemental O_2_. The effectiveness of preconditioning protocols was affected by ventilation. (**A**) Area at risk expressed as a percentage of the left ventricle. *N* number indicated in white. (**B**) Area of infarct expressed as a percentage of the area at risk. *N* number indicated in white. By two‐way anova, there was a significant effect of oxygenation (*P* < 0.01), treatment (*P* < 0.001) and interaction (*P* < 0.001). **P < 0.01 ***P < 0.001 compared to relevant controls. †*P* < 0.05 compared to room air control.

Mice ventilated with either room air or 100% O_2_ were subject to 40‐min. coronary ischaemia followed by 120‐min. reperfusion, after which the ischaemic area at risk and infarct area were measured. IPC was applied directly to the heart for 5 min. or remotely to the hindlimb for three cycles of 2.5‐, 5‐ or 10‐min. ischaemia separated by 5‐min. reperfusion (Fig. 1). Bradykinin (40 μg/kg) was injected i.v. as a positive control for a pharmacological preconditioning mimetic that does not depend on hypoxia to induce cardioprotection. The area at risk was similar in all groups (Fig. 2A) (see Fig. S2 for representative images of TTC‐stained heart slices).

Infarct size was significantly smaller in mice breathing O_2_ compared to those breathing room air (*P* < 0.01 for an effect of ventilation method by two‐way anova,* P* < 0.05 when comparing control groups only by Bonferroni correction) (Fig. 2B). RIPC induced using either 2.5‐ or 5‐min. cycles was cardioprotective in mice breathing room air, but not supplementary O_2_ (Fig. 2B). An RIPC protocol using a longer cycle time of 10 min. was ineffective under both ventilation strategies (Fig. 2B). Reasoning that supplementary O_2_ may interfere with the induction of hypoxia/ischaemia in the limb, we tested direct cardiac ‘IPC’ and found that this was able to reduce infarct size in room air, but was similarly ineffective under O_2_ (Fig. 2B). In contrast, pharmacological protection using bradykinin was able to reduce infarct size in both settings (Fig. 2B).

Heart rate was measured at selected time‐points during the experiment. There was a small but significant decrease in heart rate over the course of the experiment (*P* < 0.01), but there were no significant effects of treatment on heart rate over this time period (Fig. S1). When analysing individual time‐points, heart rate at baseline (measured 5 min. before ischaemia) was slightly elevated (*P* < 0.05) in the mice breathing O_2_ that had been treated with bradykinin, as anticipated. The only other time‐point to show a significant difference was the 15‐min. time‐point, when heart rate was lower in the RIPC 2.5 min. group on room air. On average, the heart rate in all mice receiving oxygen was consistently lower at each time‐point than the heart rate of those breathing room air at all time periods, averaging 6.0 ± 1.6 bpm lower, although this was not statistically significant by two‐way anova (*P* = 0.31).

**Figure 3:**
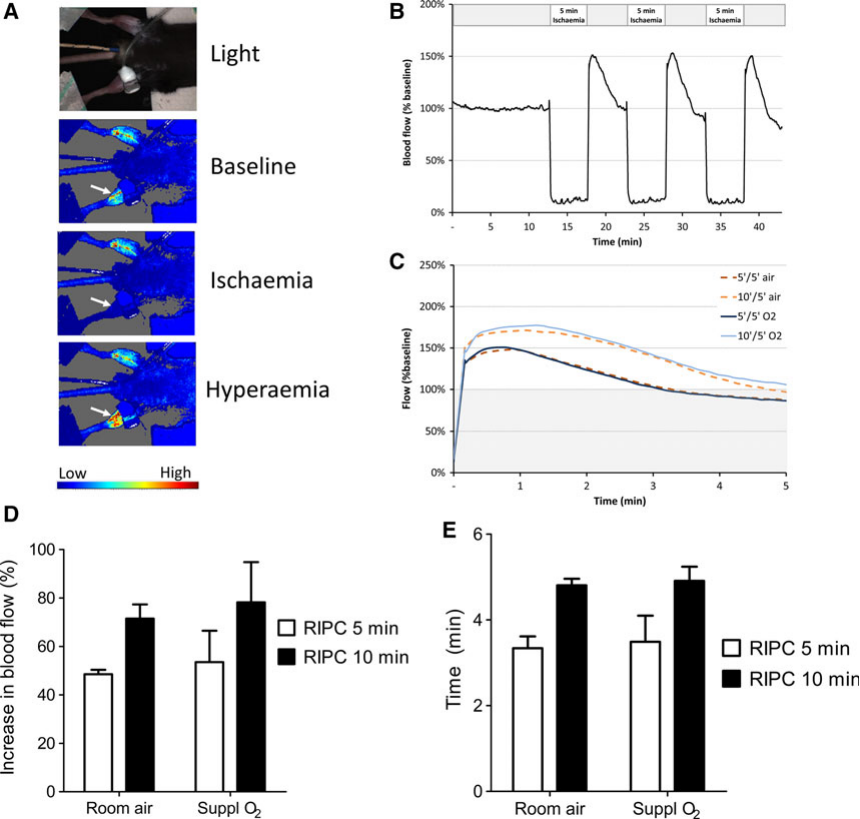
Blood flow (reactive hyperaemia) in the hindlimb measured by laser speckle contrast imaging. (**A**) Colour image of blood flow in the hindlimb, demonstrating cessation of blood flow after cuff inflation (‘ischaemia’), and hyperaemic response after release (white arrows). (**B**) Chart of blood flow in RIPC limb during a representative experiment, relative to baseline of 100%. (**C**) Average blood flow during reperfusion phase, averaged over three phases and *n* = 3 mice. (**D, E**) Maximum increase in blood flow tissue pO2 (mmHg) (**D**) and time to recover to baseline (**E**) during the reperfusion phase of RIPC. By two‐way anova, RIPC time affected the increase in blood flow (*P* < 0.05), although no differences were significant by Bonferroni *post hoc* test. *N* = 3 mice per group.

We hypothesized that ventilation with supplementary oxygen might alter peripheral vasomotor tone, thereby affecting reactive hyperaemia, which is believed to be involved in the mechanism of RIPC 9. For these and subsequent experiments, we focussed on 5‐*versus* 10‐min. limb ischaemia, with ventilation by either room air or supplementary O_2_. Laser speckle contrast tissue imaging was used to measure blood flow in the limb with high sampling frequency. Static images illustrate the complete cessation of blood flow in the ischaemic limb during RIPC and hyperaemia following reperfusion (Fig. 3A and B). We measured the degree of reactive hyperaemia during the three reperfusion phases of RIPC. As the response was virtually identical in each of the three cycles, these were averaged together for each mouse (Fig. 3B and C). The peak hyperaemic response was significantly higher after 10‐min. occlusion than after 5 min. (*P* = 0.05), but was not affected by ventilation protocol (Fig. 3C and D). Similarly, the length of time required for the blood flow to return to baseline (that is, 100% flow) was longer after 10‐min. occlusion than 5 min., but this was similarly unaffected by ventilation protocol (Fig. 3C and E). In separate mice, changing ventilation from air to O_2_ did not significantly alter hindlimb blood flow (*N* = 3, *P* = 0.25). Blood flow was also measured in the non‐preconditioned limb and was found to increase by 8.8 ± 1.1% after release of the cuff, suggesting a systemic response; however, this was not significantly different in any of the groups (data not shown).

**Figure 4:**
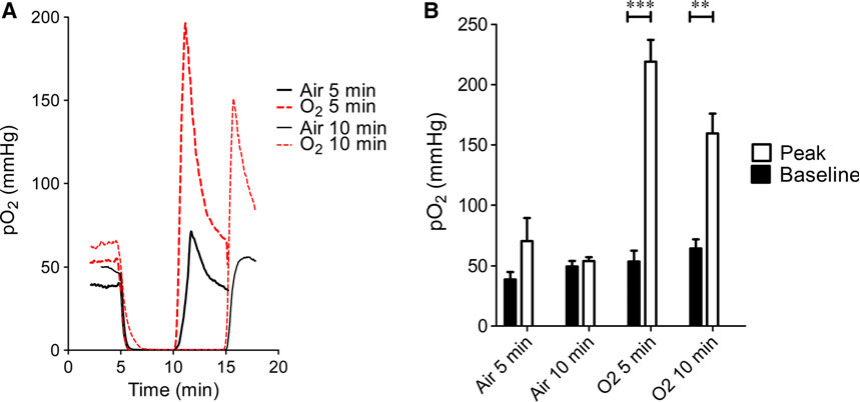
(**A**) Average limb tissue pO2 (mmHg) from baseline to the end of the first reperfusion phase (*N* = 3 mice per group). (**B**) Average limb tissue pO2 at baseline and the peak during the reperfusion phase of RIPC. Peak and baseline differed significant by paired two‐way anova. **P < 0.01, ***P < 0.001. *N* = 3 mice per group.

To test the hypothesis that supplementary oxygen affected the time taken to achieve muscle hypoxia, we measured limb pO_2_ with a fine, phosphorescent tissue sensor. The average traces of pO_2_ over time demonstrate that basal muscle pO_2_ was slightly elevated in mice ventilated with supplementary oxygen (red traces, Fig. 4A). pO_2_ rapidly decreased to <1 mmHg within a minute of limb occlusion, and this drop was very similar in mice breathing room air and supplementary oxygen. After reperfusion, pO_2_ increased rapidly, exhibiting a hyperaemic overshoot which was much greater in mice breathing supplementary oxygen (Fig. 4A and B). Thus, the total time during which tissue was hypoxic was not altered by different ventilation strategies. Interestingly, after the cuff was deflated, pO_2_ returned to baseline ~10 times more slowly (30–75 sec.) than blood flow returned to baseline (3–7 sec., data not shown).

## Discussion

In these studies, we set out to determine whether supplemental oxygen would affect infarct size in mice subject to ischaemia and reperfusion. In two separate groups of mice (control and vehicle), ventilation with supplemental oxygen was found to reduce infarct size. Furthermore, a standard protocol of three cycles of 5‐min. RIPC was unable to induce further protection. This was not because the mice were at a ‘ceiling’ of cardioprotection because the pharmacological preconditioning agent, bradykinin, was able to induce additional cardioprotection. Longer or shorter periods of limb ischaemia were similarly ineffective at inducing RIPC in supplemental oxygen, as was direct cardiac IPC, despite it being effective in room air. These results led us to hypothesize that alterations in limb oxygenation or in the restoration of limb blood flow during the reperfusion phase (perhaps secondary to peripheral vasoconstriction) could explain the inability to induce protection. This possibility was supported by the observation that cycles of RIPC with 10‐min. ischaemia duration were ineffective at inducing cardioprotection even in room air, which might suggest that limb reperfusion was inadequate after prolonged ischaemia. However, when blood perfusion or limb pO_2_ was directly and precisely measured by laser speckle contrast imaging and using an intramuscular pO_2_ sensor, no alterations in the induction of tissue hypoxia, total hypoxia duration, or restoration of blood flow and reactive hyperaemia were found that could explain the observations.

### Why are mice ventilated with supplemental O_2_ protected?

We considered several possible reasons for mice breathing O_2_ having smaller infarcts. Higher oxygen saturation can induce cardiac bradycardia 10, and heart rate is known to influence infarct size. However, we observed no significant effect of ventilation strategy on heart rate. On average, the heart rate in mice receiving oxygen was 6.0 ± 1.6 bpm (~1%) less than those receiving room air, but this small difference seems unlikely to account for the significant protection. A mechanism involving redistribution of coronary blood flow to the ischaemic region *via* coronary collaterals is not possible, because the strain of mice used here lack significant coronary collateralization 11. It may be argued that increased pO_2_ improves oxygenation of peri‐infarct regions *via* simple diffusion into the ischaemic periphery, but it must be remembered that the tissue pO_2_ gradient in the heart is extremely high due to the high oxygen consumption rates of cardiomyocyte mitochondria 12.

Transient exposure to hyperoxia lasting at least 120 min. has been shown to mimic preconditioning, reducing infarct size in rats subsequently subjected to ischaemia and reperfusion 13, although the mechanism was not established. In humans, hyperoxia has been shown to increase parasympathetic influence on the heart 10. RIPC is believed to induce cardioprotection *via* two parallel pathways, one involving the release of a humoural factor and the other *via* parasympathetic activation 14, 15, 16, 17. Therefore, parasympathetic activation by hyperoxia could potentially explain why hearts are protected and not amenable to further protection by RIPC. In this scenario, bradykinin is capable of delivering additional cardioprotective benefit *via* a direct signalling mechanism.

Hyperoxia also appears to reduce infarct size in other organs. For example, hyperoxia during cerebral ischaemia in rats has been shown to slow infarct development as well as decreasing ultimate infarct size 18, 19, 20. Hyperoxia in this model did not alter cerebral blood flow 18.

### Why is ischaemic conditioning ineffective when breathing supplemental O_2_?

The explanation for the ineffectiveness of IPC and RIPC in mice breathing supplemental O_2_ is unclear. Despite the activation of a protective mechanism in these mice, there is clearly room for additional protection, as demonstrated by the use of bradykinin. This suggests that the inability to induce protection is related either to the inability to produce sufficient ischaemia under these conditions or to the inability of ischaemia to activate protective signalling pathways. Reactive hyperaemia is believed to be important for the induction of protective signalling pathways by RIPC 9. As oxygen status can cause dramatic redistribution of blood flow between the periphery and central organs 21, 22, we tested that hypothesis that limb perfusion or reactive hyperaemia was decreased when ventilated with oxygen. However, we observed no effect of ventilation strategy on resting hindlimb blood flow or on the ability to reperfuse the limb, as demonstrated by a robust hyperaemic response during the reperfusion phase of RIPC.

RIPC applied to the lower limbs increases coronary blood flow in pigs 23, and RIPC also transiently increases coronary blood flow in healthy human beings, *via* a hyperaemic response during early reperfusion 24. Thus, the hyperaemia of RIPC appears to be a systemic response. We are unable to measure coronary blood flow in the mouse but we detected a consistent increase in blood flow in the non‐RIPC limb following the RIPC procedure. Again, however, this response was unaltered by oxygen status.

### How important is the ischaemia duration during RIPC?

In room air, 2.5‐ and 5‐min. periods of ischaemia during RIPC were protective, but 10‐min. periods were not. Identical results were observed by Botker's group using a model in which RIPC was applied to mice *in vivo*, before hearts were removed, perfused on a Langendorff apparatus, and infarcted *ex vivo* 25. This would suggest that careful optimization of the ischaemic duration in patients is important. The reason for loss of protection with 10‐min. protocol is not clear. After 10 min., the vessels of the hindlimb remained patent and limbs reperfused well – indeed, the hyperaemic response was significantly greater than after 5 min. Limb pO_2_ was restored to normal with similar kinetics after 5‐ and 10‐min. ischaemia. One possibility is that longer durations of limb ischaemia cause the release of an ‘anti‐protective’ factor that counteracts the benefits of RIPC. Extensive further work would be required to confirm this hypothesis, for example by measuring plasma levels of parameters such as proinflammatory cytokines.

We conclude that ventilation with room air or supplemental oxygen can have a major influence on final infarct size in mice. All tested remote and direct IPC protocols were ineffective when using supplemental oxygen, despite pharmacological protection remaining effective. Ventilation strategy should be taken into consideration when designing infarction experiments in mice. Furthermore, this variable may potentially be important in the design of clinical studies and may explain, in part, some of the neutral results to date. However, clinical studies will be required to confirm the effect of ventilation on RIPC, and these should also be designed to take into account the fact that 30–50% FiO2 is typically used during mechanical ventilation of patients under anaesthesia.

## Conflict of interest

The authors declare that they have no conflict of interest.

## Supporting information


**Fig. S1.** Average heart rate at various time‐points of surgery.

**Fig. S2**. Representative images of TTC‐stained hearts from each treatment group.
